# Lewy Body Dementia Association’s Research Centers of Excellence Program: Inaugural Meeting Proceedings

**DOI:** 10.1186/s13195-019-0476-1

**Published:** 2019-03-13

**Authors:** Bethany Peterson, Melissa Armstrong, Douglas Galasko, James E. Galvin, Jennifer Goldman, David Irwin, Henry Paulson, Daniel Kaufer, James Leverenz, Angela Lunde, Ian G. McKeith, Andrew Siderowf, Angela Taylor, Katherine Amodeo, Matt Barrett, Kimiko Domoto-Reilly, John Duda, Stephen Gomperts, Neill Graff-Radford, Samantha Holden, Lawrence Honig, Daniel Huddleston, Carol Lippa, Irene Litvan, Carol Manning, Karen Marder, Charbel Moussa, Chiadi Onyike, Fernando Pagan, Alexander Pantelyat, Victoria Pelak, Kathleen Poston, Joseph Quinn, Irene Richard, Liana S. Rosenthal, Marwan Sabbagh, Douglas Scharre, Sharon Sha, Holly Shill, Yasar Torres-Yaghi, Tina Christie, Todd Graham, Ian Richards, Mike Koehler, Brad Boeve

**Affiliations:** 1 Lewy Body Dementia Association, Lilburn, USA; 2 University of Florida, Gainesville, USA; 3 University of California, San Diego, USA; 4 Florida Atlantic University, Boca Raton, USA; 5 Rush University Medical Center, Chicago, USA; 6 University of Pennsylvania, Philadelphia, USA; 7 University of Michigan, Ann Arbor, USA; 8 University of North Carolina, Chapel Hill, USA; 9 Cleveland Clinic, Cleveland, USA; 10 Mayo Clinic campus, 200 1st Street SW, Rochester, MN 55905 USA; 11 Newcastle University, Newcastle, UK; 12 University of Rochester, Rochester, USA; 13 University of Virginia, Charlottesville, USA; 14 University of Washington, Seattle, USA; 15 Massachusetts General Hospital, Boston, USA; 16 Mayo Clinic campus, Jacksonville, FL USA; 17 University of Colorado, Aurora, USA; 18 Columbia University, New York City, USA; 19 Emory University, Atlanta, USA; 20 Thomas Jefferson University, Philadelphia, USA; 21 Georgetown University Medical Center, Washington, D.C., USA; 22 Johns Hopkins University, Baltimore, USA; 23 Stanford University, Stanford, USA; 24 Oregon Health & Science University, Portland, USA; 25 Cleveland Clinic Las Vegas, Las Vegas, USA; 26 The Ohio State University, Columbus, USA; 27 Barrow Neurological Institute, Phoenix, USA

**Keywords:** Lewy body dementia, Lewy Body Dementia Association, Parkinson’s disease dementia

## Abstract

The first Lewy Body Dementia Association (LBDA) Research Centers of Excellence (RCOE) Investigator’s meeting was held on December 14, 2017, in New Orleans. The program was established to increase patient access to clinical experts on Lewy body dementia (LBD), which includes dementia with Lewy bodies (DLB) and Parkinson’s disease dementia (PDD), and to create a clinical trials-ready network. Four working groups (WG) were created to pursue the LBDA RCOE aims: (1) increase access to high-quality clinical care, (2) increase access to support for people living with LBD and their caregivers, (3) increase knowledge of LBD among medical and allied (or other) professionals, and (4) create infrastructure for a clinical trials-ready network as well as resources to advance the study of new therapeutics.

## Introduction

The first Lewy Body Dementia Association (LBDA) Research Centers of Excellence (RCOE) Investigator’s meeting was held on December 14, 2017, in New Orleans. The program was established to increase patient access to clinical experts on Lewy body dementia (LBD), which includes dementia with Lewy bodies (DLB) and Parkinson’s disease dementia (PDD), and to create a clinical trials-ready network. Mayo Clinic, Rochester MN, was designated the coordinating center following a competitive application process. The 24 centers were selected by an application and peer-review process.

## Background

The top national research priority for LBD is to initiate clinical trials to address symptoms that have the greatest impact on patient function and caregiver burden. LBDA’s RCOE program establishes infrastructure to run multi-center studies with expertise, centralization, and standardized data collection. The program will yield a well-characterized, nationwide group of LBD patients for rapid recruitment into therapeutic trials.

## Aims of the program

The major aims are to:

- Increase access to high-quality clinical care
- Increase access to support for people living with LBD and their caregivers
- Increase knowledge of LBD among medical and allied (or other) professionals
- Create infrastructure for a clinical trials-ready network as well as resources to advance the study of new therapeutics.

Four working groups (WG) were created to pursue the LBDA RCOE aims. WG chairs (JEG, JGG, HP, MJA, DK, DI, JBL, AS) were selected; all PIs self-selected in which WGs they would participate. Each WG had several goals to achieve for the meeting, summarized below.

## Working groups

### Clinical care and professional education WG

WG goals are to ascertain best practice clinical guidelines and identify opportunities for professional LBD education. The group discussed strategies to solicit feedback from experts and other stakeholders on diagnosis and symptom management. Diagnosis using the fourth consensus report of the DLB consortium was emphasized [1], but challenges regarding its widespread implementation remain. Important clinical milestones in LBD management include referrals to allied health therapies, utilization of community resources, and entry into skilled nursing and long-term care facilities. Examples of LBD research and clinical service toolkit development such as the DIAMOND-Lewy research program may help inform the LBDA RCOE efforts [2].

Increasing awareness and disseminating knowledge on LBD to healthcare professionals remains a critical priority. Key target audiences include (a) healthcare professionals such as physicians, advanced practice providers, and trainees practicing in Neurology, Psychiatry, Geriatric Psychiatry, Geriatrics, Sleep Medicine, Cardiology, Urology, Gastroenterology, and Palliative Care; those practicing in Psychology and Neuropsychology; and those practicing in long-term care settings; (b) allied health professionals (nurses, social work, physical, occupational and speech therapy, nutrition, arts/music therapy, chaplaincy, and hospice); and (c) healthcare workers in specific environments, including hospitals, assisted living facilities, long-term care facilities, and adult day programs.

### Community education and support WG

WG goals are to develop core themes and materials, and identify opportunities for community education, assistance for support groups, and best practices for caregiver education and support for non-pharmacological approaches to behavioral changes in LBD.

The WG surveyed 23 RCOE sites. Ten sites (43%) already hosted LBD-specific support groups, most commonly targeting caregivers alone or caregivers and people with LBD together. These groups were typically led by either clinical staff or former caregivers and met on a monthly basis. Sites preferred either in-person or mixed in-person/virtual monthly support groups led by clinical staff. Most sites favored targeting caregivers and/or early-stage people with LBD; some sites suggested support groups for moderate- and late-stage people with LBD. Trained facilitators were described as essential.

LBDA RCOE sites commonly educate people with LBD and their families with LBDA-produced materials, the LBD booklet from the National Institutes of Health, and materials produced by the individual sites.

### Clinical trial design and optimization WG

WG goals are to identify ongoing or planned therapeutic trials in LBD, identify and address key gaps in clinical assessment tools for use in diagnosis of LBD and clinical trials, identify current or emerging biomarkers relevant for LBD clinical trials, and develop strategies for implementation. Collectively, the LBDA RCOE network is estimated to see > 2600 new patient visits (~ 1300 DLB and ~ 1300 PDD) and > 7,500 return visits (~ 3000 DLB and ~ 4500 PDD) annually. The majority of sites are very active in clinical trials for LBD and related disorders, with 16 centers participating in ≥ 1 clinical trial for LBD and collectively enrolling ~ 430 LBD patients in the past year. Furthermore, 21 sites participated in ≥ 2 and 18 sites are active in ≥ 5 clinical trials for related neurodegenerative disorders.

The RCOE investigator survey and discussion revealed a strong interest in trials focused on both symptomatic therapies and potential disease-modifying agents. Prioritized targets for non-pharmacological interventions included caregiver-stress, agitation, and motor function limitations. One critical need is to identify and develop clinically meaningful DLB clinical outcome measures, including ways to account for and/or quantify the effects of cognitive fluctuations. Emerging biomarkers were classified on their potential for diagnostic and/or prognostic use based on review of the current state of the science. High-yield applications of diagnostic biomarkers for LBD included identifying prodromal disease, differentiation of LBD from other dementias, and stratification of biologically meaningful subgroups of DLB patients such as those with significant AD co-pathology or those harboring a glucocerebrosidase gene mutation. There was strong interest in the need for alpha-synuclein-specific markers; however, it was felt that current assays require further development and validation. Other top priorities identified included tissue-validation and standardization of imaging and fluid biomarkers, and the development of an infrastructure for sharing biomarker specimens and data that will be obtained in future clinical trials to the research community at large.

### Industry engagement WG

WG goals are to determine general principles for interacting with industry partners and determine requirements for industry-sponsored clinical trials, including data and sample sharing and publication plan, contract and budget development, and protocol optimization. The WG serves as a central resource for exploring collaborations between the RCOE and industry on LBD clinical trials.

To maximize efficiencies in collaborations with industry, this working group has proposed an accessible, centralized data resource on the clinical trial capabilities of all RCOE sites. This will facilitate exploring clinical research collaborations and expedite site selection for industry-sponsored trials. Each site will determine its unique clinical trial capacity, its ability to recruit people with LBD for trial enrollment, and its testing capabilities. The RCOE program will strive to use a standardized contract and a central institutional review board. In parallel with the effort to build the infrastructure, this working group will provide guidance on establishing expert panels to advise clinical trial sponsors.

## Conclusions

The LBDA established the RCOE program to accelerate access to high-quality care and support, increase disease awareness among healthcare providers, and advance the study of symptomatic and disease-modifying treatments. As the RCOE program grows and evolves, it will continue to share its successes and lessons learned with the scientific community. The next RCOE Principal Investigator’s meeting will be in June 2019.

## Abbreviations

AD: Alzheimer’s disease
DLB: Dementia with Lewy bodies
LBD: Lewy body dementia
LBDA: Lewy Body Dementia Association
PDD: Parkinson’s disease dementia
RCOE: Research Centers of Excellence
WG: Working group
